# Medication-induced acute esophageal necrosis: a case report

**DOI:** 10.1186/s13256-016-1043-z

**Published:** 2016-09-29

**Authors:** Lauri Pautola, Tapio Hakala

**Affiliations:** North Karelia Central Hospital, Tikkamäentie 16, 80210 Joensuu, Finland

**Keywords:** Acute esophageal necrosis, Gurvits syndrome, Olanzapine, Clozapine

## Abstract

**Background:**

Acute esophageal necrosis or Gurvits syndrome is a rare clinical condition characterized by necrotic esophageal mucosa with an abrupt end at the gastroesophageal junction. Its etiology is multifactorial, but mainly related to low-flow states. We describe a case in which a patient accidentally took the wrong medication, with clozapine and olanzapine most probably being the cause of his subsequent acute esophageal necrosis. This situation is, to the best of our knowledge, unprecedented in the medical literature.

**Case presentation:**

A 65-year-old Finnish male patient with schizoaffective disorder accidentally took another patient’s medication, including clozapine 300 mg, olanzapine 30 mg, teofyllamine 200 mg, warfarin 5 mg, and potassium chloride 1 g. He arrived at our hospital for a routine examination 6 h after the incident. At hospital he started to vomit brownish liquid and had tachycardia and fever. Gastroparesis was found. An endoscopy revealed necrotic esophageal mucosa that was typical for Gurvits syndrome. A computed tomography scan showed an edematous esophagus and raised suspicion of a proximal jejunal obstruction. A laparotomy was performed but only healthy paralytic bowel was found. Our patient healed uneventfully within a week.

**Conclusions:**

There are analogous case reports describing ischemic colitis associated with the use of clozapine and olanzapine, but none describing the same for the other medications our patient took. We believe that in this case clozapine and olanzapine caused acute esophageal necrosis and this possibility should be taken into account when treating patients with acute ischemic enteropathy.

## Background

Acute esophageal necrosis (AEN), also referred as Gurvits syndrome [1], is a rare clinical entity with multifactorial etiology. It has been associated with low-flow states of diverse backgrounds, including corrosive injury by gastric secretions, upper gastrointestinal obstruction, and malnutrition. The most common clinical presentation is upper gastrointestinal bleeding. Typically, gastroscopy reveals necrotic mucosa at the inferior esophagus with an abrupt change to healthy tissue at the gastroesophageal junction. The true prevalence of the syndrome is unknown but it has been estimated to affect up to 6 % of patients admitted to hospital for upper gastrointestinal bleeding [2]. Because of the otherwise debilitated physical state of patients, a 32 % mortality rate has been reported [1].

So far there have been no reports in the medical literature about medication-induced AEN. We describe a case in which accidentally taken medication was most probably the etiological factor. In this case, the drugs most suspected of causing the AEN were clozapine and olanzapine. Both are antipsychotic drugs with neurological effects that mainly act on the dopaminergic pathways but that also have anticholinergic, antiserotonergic, and antihistaminic effects. Reported gastrointestinal side effects include obstipation, hypersalivation, ileus, and hypomotility, occasionally leading to necrotizing colitis which can be life-threatening. Most reports are about clozapine, but similar effects have also been described with olanzapine [3, 4].

## Case presentation

Our patient was a 65-year-old Finnish male with schizoaffective disorder who was living in a local nursing home. His regular medication included chlorprothixene 50 mg twice a day, valproic acid with morning dose of 500 mg and evening dose of 1000 mg, propranolol 50 mg three times a day, and hydrochlorothiazide 12.5 mg. Propranolol and hydrochlorothiazide were prescribed previously to treat medication-induced peripheral edema and tremor. Our patient accidentally received the medication of another patient: clozapine 300 mg, theophylline 200 mg, olanzapine 30 mg, warfarin 5 mg, and 1 g of potassium chloride. Four hours after the initial dose he arrived at our emergency room for a clinical examination.

**Fig. 1:**
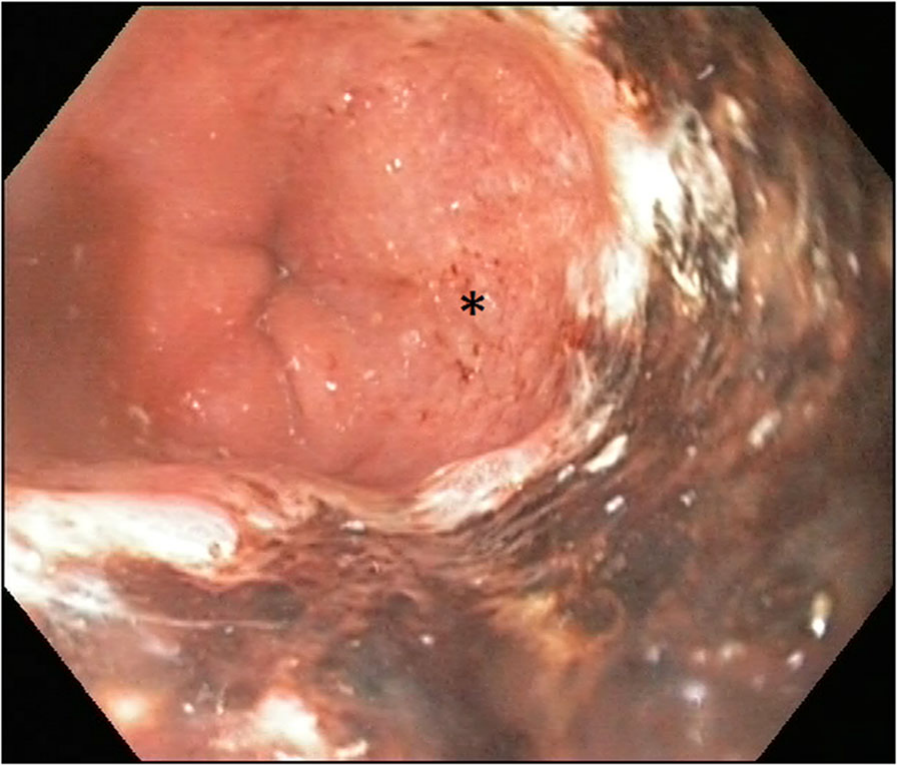
Necrotic esophageal mucosa with an abrupt change to healthy gastric mucosa at the gastroesophageal junction* healthy gastric mucosa

**Fig. 2:**
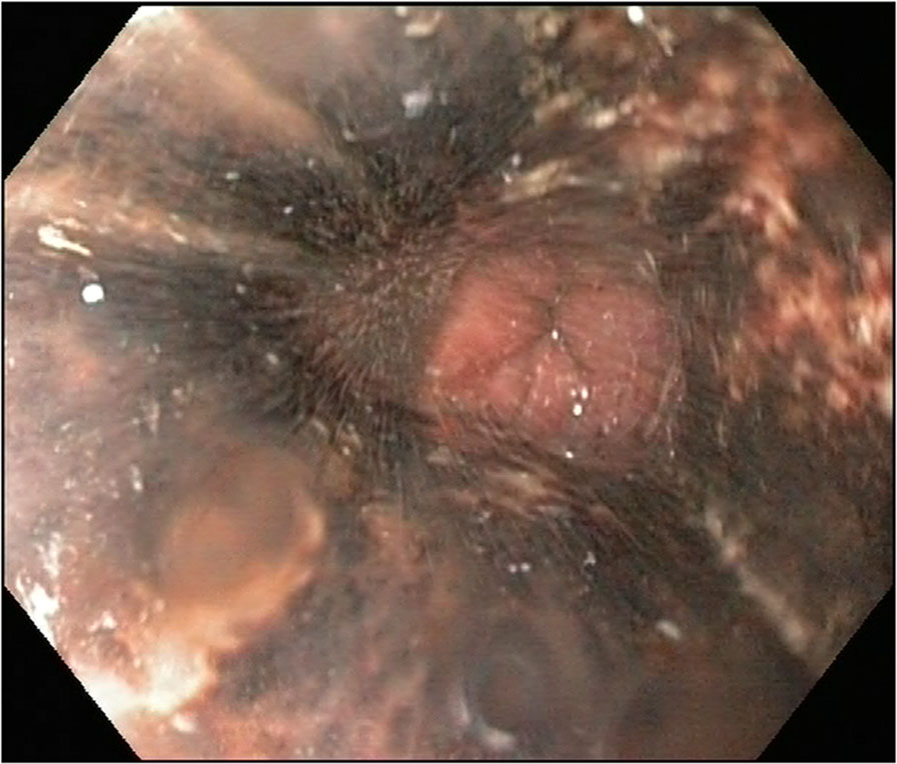
Necrotic esophageal mucosa

**Fig. 3:**
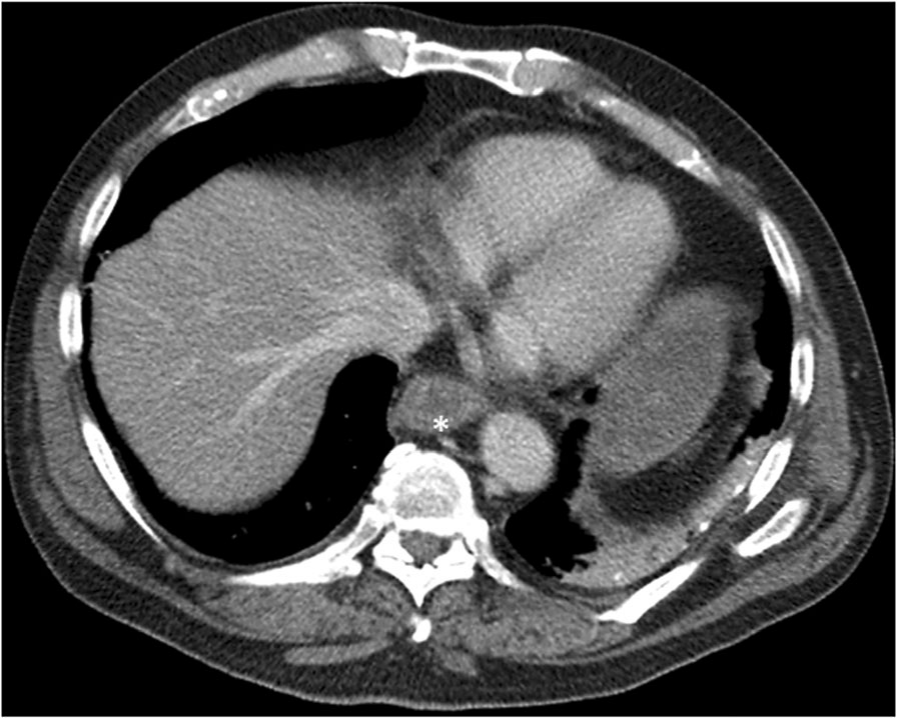
Computed tomography scan taken 12 hours after the patient ingested the wrong medication, showing an edematous distal esophagus (*) but no extraluminal air or inflammation

The first clinical examination took place 6 h after our patient took the incorrect medication. This examination was unremarkable and demonstrated that he had a soft non-tender abdomen. Results from an electrocardiogram were normal and there was no tachycardia or fever. Blood tests revealed a normal leucocyte level, an international normalized ratio of 1.0, minor thrombocytopenia (127 × 10^9^/l), and a normal C-reactive protein level. Two hours later, our patient started to complain about upper abdominal pain and vomited brown liquid several times. An abdominal X-ray revealed gastric stasis. The next morning (14 h after taking the wrong medication) a gastroscopy was performed, which revealed necrotic esophageal mucosa starting 25 cm from the upper incisor teeth that abruptly ended at his gastroesophageal junction (Figs. 1 and 2). The ventricle was extremely dilated because of the presence of air but only a minimal amount of liquid was present. A computed tomography (CT) scan was performed, which showed an edematous esophagus (Fig. 3) but no perforation. There was a sharp twist in the mesentery of his proximal small bowel with minor changes in the diameter of his bowel lumen.

Our patient was admitted to our surgical ward with a nasogastric tube in place. Antimicrobial medication with cefuroxime 1.5 g  three times a day was started. The next day our patient was feeling worse, and his C-reactive protein concentration had risen to 163 mg/l. An exploratory laparotomy was decided upon, partly based on the suspicion of proximal small bowel obstruction. During the operation no signs of any obstructing lesion were found and his bowel seemed completely healthy. A repeat gastroscopy was performed the following day. The esophageal mucosa had started to drop off and there was a healthy muscular layer underneath.

Our patient healed uneventfully with conservative treatment and was admitted back to nursing home on the seventh day. A control-endoscopy was performed 2 months later that showed a completely healed esophageal wall with no signs of stricture or scarring.

## Discussion

Although the etiology of AEN is mostly unknown, we believe that medication – clozapine and olanzapine in particular – was the causative factor in this case. First, our patient was feeling completely healthy before accidentally taking the wrong medication. Second, his symptoms progressed with a logical sequence and timing after the initial dose, as did his biochemical parameters (Table 1). The clinical presentation was typical for AEN compared to what has previously been described in the literature and thus we consider the diagnosis certain [1].

**Table 1:** Progression of vital and inflammatory parameters after taking the wrong medication

Time	Heart rate (/min)	Body temp (°C)	White blood cell count (10^9^/l)	C-reactive protein (mg/l)
6 h	108	38.6	14.1	3
Day 1	100	37.4	8.8	162
Day 2	85	37.3	11.5	193
Day 3	62	36.9	8.6	130
Day 4	73	36.6	6.5	70
Day 5	80	36	7.5	48
Day 6	70	36.1	8.8	28

Of the medications taken, clozapine is known to cause various gastrointestinal symptoms, including ischemic colitis and there are case reports describing the same for olanzapine [2–4]. On the contrary, we could not find any reports in this context on theophylline, warfarin, or potassium chloride.

## Conclusions

Antipsychotic medications should be kept in mind as a possible etiology when treating patients with AEN and other ischemic enteropathies.

## Abbreviations

AEN: Acute esophageal necrosis
CT: Computed tomography
